# Retraction: Integrated multi-omics analysis reveals complement component 3 as a central driver of immune dysregulation in polycystic ovary syndrome

**DOI:** 10.3389/fendo.2026.1898058

**Published:** 2026-06-05

**Authors:** Frontiers Editorial Office

The journal retracts the 03 March 2026 article cited above.

Following publication, the authors contacted us with concerns regarding their article. During investigation, further concerns were raised regarding the validity of the data. The authors failed to provide the raw data or a satisfactory explanation during the investigation, which was conducted in accordance with Frontiers’ policies. Given the concerns, and the lack of raw data, the editors no longer have confidence in the findings presented in the article.

This retraction was approved by the Chief Editors of Frontiers in Endocrinology and the Chief Executive Editor of Frontiers. The authors received communication regarding the retraction and had a chance to respond. This communication has been recorded by the publisher.

